# Association between obesity and COVID-19 mortality and length of stay in intensive care unit patients in Brazil: a retrospective cohort study

**DOI:** 10.1038/s41598-022-17197-w

**Published:** 2022-08-12

**Authors:** Vitor Barreto Paravidino, Tatiana Henriques Leite, Mauro Felippe Felix Mediano, Rosely Sichieri, Gulnar Azevedo e Silva, Victor Cravo, Alex Balduino, Emmanuel Salgueiro, Bruno Adler Maccagnan Pinheiro Besen, Rodrigo de Carvalho Moreira, Carlos Eduardo Brandão, Danilo Cosme Klein Gomes, Cinthia Almeida Guimarães Assemany, Pedro Cougo

**Affiliations:** 1 Department of Epidemiology, Institute of Social Medicine, State University of Rio de Janeiro, Rio de Janeiro, RJ Brazil; 2 Department of Physical Education and Sports, Naval Academy - Brazilian Navy, Rio de Janeiro, RJ Brazil; 3 Evandro Chagas National Institute of Infectious Disease, Oswaldo Cruz Foundation, Rio de Janeiro, RJ Brazil; 4 Department of Research and Education, National Institute of Cardiology, Rio de Janeiro, RJ Brazil; 5 Hospital Vitória e Samaritano Barra, Américas Serviços Médicos, UnitedHealth Group Brazil, Rio de Janeiro, RJ Brazil; 6 Clinical Research, Research and Education Institute, UnitedHealth Group Brazil, Rio de Janeiro, RJ Brazil; 7 Hospital das Clínicas HCFMUSP, Faculdade de Medicina, Universidade de São Paulo, São Paulo, SP Brazil; 8 Department of Standard Care - Amil, UnitedHealth Group, São Paulo, Brazil

**Keywords:** Epidemiology, Infectious diseases

## Abstract

The present study aimed to evaluate the association between obesity and COVID-19 mortality and length of stay in ICU patients, and how these associations were modified by age groups. We performed a retrospective multicenter cohort study with data obtained from a hospital-based registry. The sample consisted of 8183 ICU hospitalized patients who tested positive for SARS-CoV-2. Cox proportional models were used to evaluate the association between BMI categories and COVID-19 mortality and generalized linear models for the length of stay in the ICU. After adjusting for confounders, those in the younger group with severe obesity had an increased risk of COVID-19 mortality compared to those with normal/overweight (HR 1.27; 95% CI 1.01–1.61). An increased risk of death was also observed for patients with underweight (HR 3.74; 95% CI 1.39–10.07). For patients aged ≥ 60 year, mild/moderate obesity was associated with reduced mortality risk (HR 0.87; 95% CI 0.78–0.97). For the age group < 60 year, the length of stay in ICU for those patients with severe obesity was 35% higher compared to the normal/overweight category (e^β^ 1.35; 95% CI 1.21–1.51). Conversely, for the survivors in the underweight category, the length of stay in ICU was 51% lower compared to the normal/overweight group (e^β^ 0.49; 95% CI 0.31–0.78). In the age group ≥ 60 year, mild/moderate obesity was associated with an increased length of stay in the ICU (*e*^β^ 1.10; 95% CI 1.01–1.21), adjusting for confounders. These findings could be helpful for health professionals to identify subgroups at higher risk for worse outcomes.

## Introduction

In March 2020, the World Health Organization declared the COVID-19 pandemic, and by May 31, 2021, over 170 million cases and nearly 4 million deaths were reported worldwide^1^. Brazil was one of the most affected countries, counting more than 555,000 deaths, in the same period^2^.

Since then, many studies have demonstrated that the elderly and people living with diabetes, cardiovascular disease, and respiratory or kidney diseases were associated with an increased risk of adverse outcomes and poor COVID-19 prognosis^3,4^. Also, there is a consistent finding that obesity is associated with worse COVID-19 outcomes^5^, with several systematic reviews and meta-analyses showing that obesity was associated with a higher risk of infection^5,6^, intensive care unit (ICU) admission^5,7–9^, severe COVID-19^5,7,8,10^, invasive mechanical ventilation^5,7,9^, and mortality^4–6,8,10,11^.

However, most meta-analyses showed a large heterogeneity for the combined effect related to the association between obesity and COVID-19 mortality, and only a few tried to explain this issue^10,12^. Two meta-analyses with metaregression suggested that age could be an effect modifier between obesity and COVID-19 mortality^10,12^, showing that increased age was associated with a decrease in the magnitude of the association. Similar results were demonstrated in another meta-analysis that conducted a subgroup analysis by age. The authors showed that among individuals younger than 60 years old, the combined odds ratio was 3.30 (95% CI 2.13–5.10), and for the elderly 1.52 (95% CI 1.05–2.19)^13^. Other studies also demonstrated that the association between BMI and adverse COVID-19 outcomes was stronger in younger individuals^14–16^.

In relation to critically ill COVID-19 patients, Dana et al. was the first study to report that in-hospital mortality was significantly lower for patients admitted to the ICU with BMI 30–39.9 kg/m^2^, compared to those with normal weight, overweight or severe obesity^17^. From other respiratory diseases, some studies also showed reduced ICU and mortality rates in overweight or obese people compared to normal BMI, suggesting the so-called ‘obesity paradox’^18–20^. Although older individuals are at a greater risk of COVID-19 mortality, and some studies observed an effect modification by age for the association between obesity and COVID-19 mortality, the interaction of obesity and age in critically COVID-19 patients is still unclear. A recent systematic review of COVID-19 in-hospital mortality concluded that obesity was only associated with mortality in studies that included fewer critical patients^21^.

Another important aspect related to COVID-19 infection is the length of stay in the ICU. Some authors have already shown that COVID-19 demands a prolonged length of stay in ICU, with the oldest patients spending the longest period^22^; however, little is known about the association between BMI status and length of stay in ICU in patients with COVID-19.

Therefore, the primary objective was to evaluate the association between obesity and mortality in patients with COVID-19 admitted to the ICU in Brazil. The secondary objective was to evaluate the association between obesity and length of stay in the ICU among the survivors and to assess the potential role of age as a modifier of these associations. We hypothesized that severe obesity would be associated with increased mortality risk and a greater length of stay in intensive care units among younger patients and associated with reduced mortality risk and length of stay in intensive care units among older patients, in comparison to normal/overweight.

## Methods

### Study design and setting

This is a retrospective multicenter cohort study of a hospital-based registry oriented to clinical and administrative purposes^23^. The registry is utilized by ICUs pertaining to a network of 32 private hospitals in Brazil in the states of São Paulo, Rio de Janeiro, Ceará, Pernambuco, and the Federal District.

### Participants’ recruitment

In this registry, patients are consecutively recruited at ICU admission and followed up until hospital discharge. For this study, we selected all patients aged ≥ 18 years, admitted to ICU with SARS-CoV2 infection confirmed by reverse transcription-polymerase chain reaction (RT-PCR), testing between March 01, 2020, and May 31, 2021. We included patients who were admitted directly from the emergency department, transferred from a hospital ward, or referred from another hospital. According to a network-wide clinical protocol, patients with COVID-19 were admitted to ICU care when oxygen delivery exceeded 4 L per minute, when invasive ventilation or face-mask non-invasive ventilation was indicated, or whenever any associated organ dysfunction was present. All patients with missing data for body weight and/or height were excluded from the analyses since BMI calculation was not possible for these patients.

### Theoretical model

**Figure 1:**
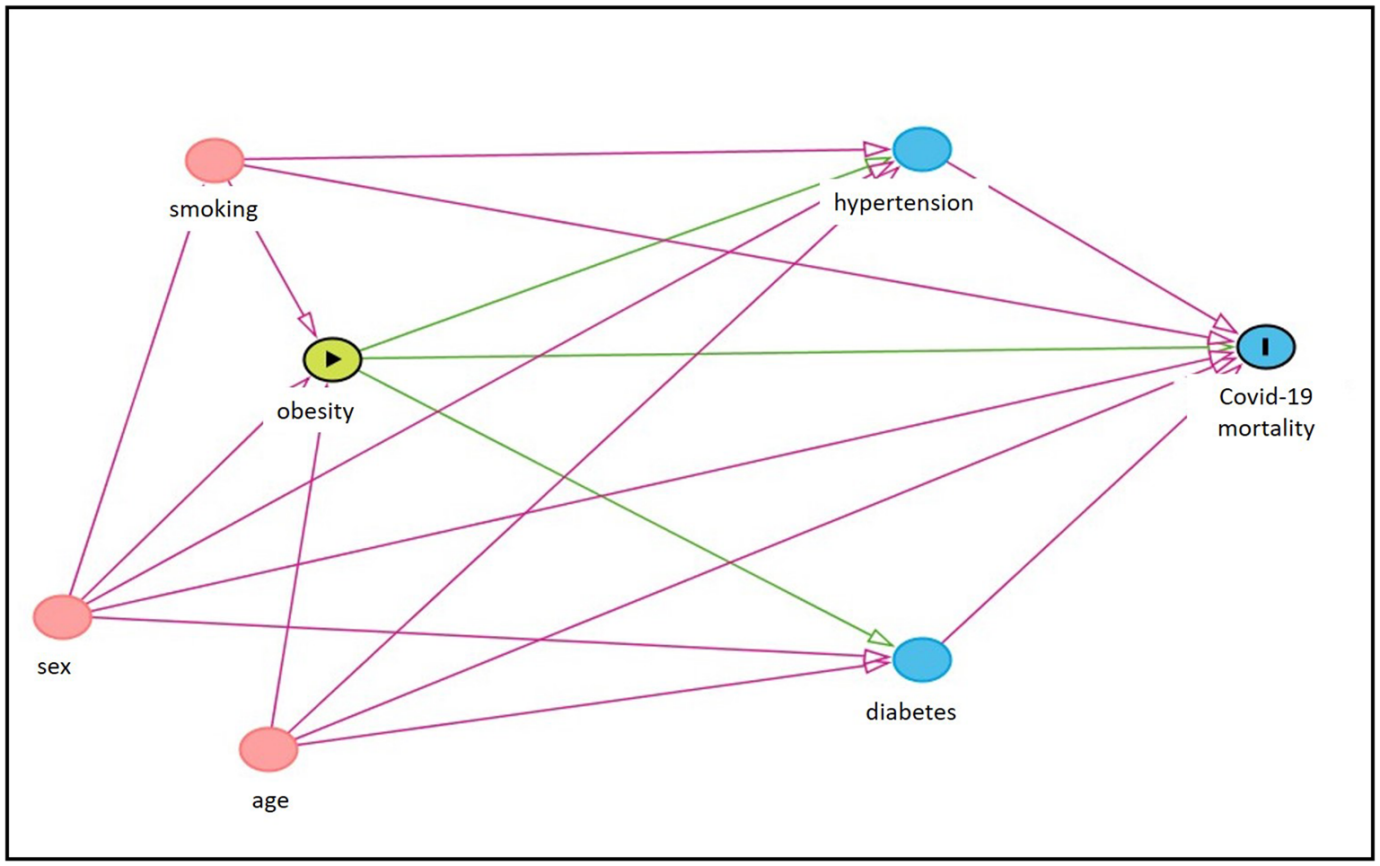
Directed Acyclic Graph presenting the potential relationship between obesity (exposure), COVID-19 mortality (outcome), and the covariates, for age groups < 60 years and ≥ 60 years.

Figure 1 shows the theoretical model examining the association between obesity (exposure) and COVID-19 mortality (outcome). The red arrows represent the back-door paths, and the green arrows represent the direct and indirect pathways between obesity and COVID-19 mortality^24^. The model postulate that smoking, sex, and age are potential common causes of both exposure and outcome. Also, hypertension and diabetes are mediator variables through the causal path between obesity and COVID-19 mortality.

### Data measurements and variables

Data collection from electronic medical records was performed daily during working days by trained nurses. Demographic variables, smoking status (yes/no), comorbidities (yes/no), and medical conditions were self-reported or informed by relatives at the moment of their admission to the hospitals. BMI was calculated as a ratio between body weight (kg) and squared height (m^2^), and categorized into four groups: underweight (BMI < 18.5 kg/m^2^), normal/overweight (18.5–29.9 kg/m^2^), mild/moderate obesity (30–39.9 kg/m^2^), and severe obesity (≥ 40 kg/m^2^). These categories were defined following a clinical rationale based on previous studies^17,25^.

The primary outcome was COVID-19 mortality during ICU stay, and the secondary outcome was the length of stay in ICU, defined as the time elapsed between ICU admission and ICU discharge, in days.

### Statistical analyses

For descriptive analysis, means and standard deviations or median and interquartile range for continuous variables and frequencies (percentage) for categorical variables were calculated for variables of interest at baseline. To compare the characteristics across BMI categories, Kruskal–Wallis test for continuous, and Chi-squared and Fisher’s exact tests for categorical variables were used.

To evaluate the association between BMI categories and COVID-19 outcomes we tested three models: (1) unadjusted model; (2) adjusted for confounders; (3) adjusted for confounders + mediators. Confounding variables were selected through a Directed Acyclic Graphs (DAG)^24^, and the minimum sufficient adjustment set for the identification of the total association of obesity on COVID-19 mortality or length of stay in the intensive care unit included the variables age, sex, and smoking (model 2), as depicted in Fig. 1. The variables set was identified using the DAGitty application^26^. Additional analysis (model 3) was performed including mediator variables (hypertension and diabetes mellitus) to investigate the direct association between the exposure and outcomes, or other possible pathways not presented in the DAG.

To evaluate the association between BMI categories and COVID-19 in-hospital mortality, Cox proportional hazards models were performed. The association between BMI categories and length of stay in ICU among the survivors was evaluated using generalized linear models, with gamma distribution and log link function. All analyses were also stratified by age below or above 60 years old.

For the elderly, sensitivity analysis grouping patients according to different BMI categories were also performed, as follows: underweight (< 22.0 kg/m^2^), normal weight (22.0–26.9 kg/m^2^), overweight (27.0–29.9 kg/m^2^), and obesity (≥ 30 kg/m^2^)^27^. Additional sensitivity analyses were performed to evaluate the effect of missing data on the variables hypertension, diabetes mellitus, and smoking status on mortality. Firstly, all missing data were considered as cases, and then, as non-cases^28^. To evaluate the possible influence of the vaccination period, we excluded older individuals (> 60 years) admitted to ICU between February 01 to May 31, 2021. This refers to the vaccination period during the study, which mostly included older adults for such period.

All analyses were performed using SAS On-demand for Academics, and the statistical significance was set at p < 0.05.

### Ethics approval

The study was analyzed and approved by the Research Ethics Committee of Pró Cardiaco Hospital with a waiver of informed consent (CAAE number: 43739321.3.0000.5533).

## Results

**Figure 2:**
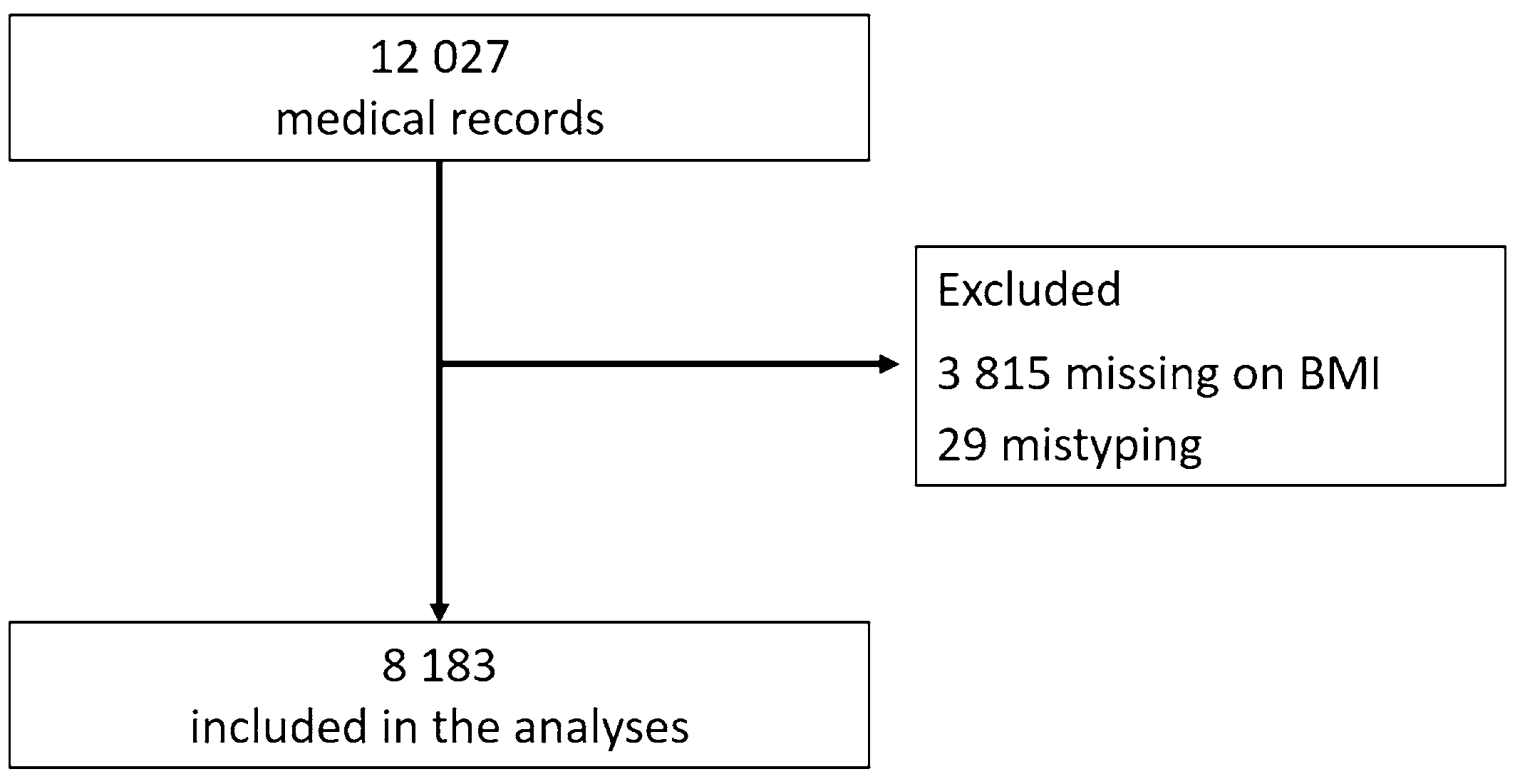
Flowchart of study participants.

Of the 12,027 medical registries in the original database, 3815 had missing data for BMI, 29 had implausible data for BMI (1 BMI value below 10 kg/m^2^ and 26 BMI values above 100 kg/m^2^), and two negative values for ICU length of stay, being excluded from the analyses. Therefore, the final sample included in the present study consists of 8183 ICU hospitalized patients (Fig. 2).

Tables 1 and 2 describe the baseline characteristics of the patients. For the 4130 patients aging < 60 years (Table 1), the mean age was 47.0 years old, with 2670 (64.7%) men, and 56.3% with some degree of obesity (BMI > 30 kg/m^2^). The most prevalent comorbidity was hypertension (55.0%), followed by diabetes mellitus (31.4%). For those aging ≥ 60 years (Table 2), the mean age was 72.0 years old, with a slight predominance of men (56.3%). The proportion of individuals with obesity was lower (35.0%) among the elderly in comparison to younger individuals. However, the prevalence of comorbidities in the older group was greater, being hypertension (77.9%) and diabetes mellitus (48.7%) the most reported. The proportion of death in the age group < 60 years was 18.7% with a median ICU length of stay of 7.4 days. In contrast, in the older age group, 43.9% of the patients died, with a median ICU length of stay of 8.8 days.

**Table 1:** Baseline characteristics of study patients aged < 60 years.

Variables	Overall (n = 4130)	BMI categories (kg/m^2^)				p-value*
		< 18.5 (n = 25)	18.5–29.9 (n = 1780)	30.0–39.9 (n = 1882)	≥ 40.0 (n = 443)	
Age (years)	47.0 (14.0)	34.0 (25.0)	48.0 (14.0)	47.0 (14.0)	42.0 (14.0)	< 0.001
**Sex**						< 0.001
Men	2670 (64.7)	12 (0.5)	1163 (43.6)	1240 (46.4)	255 (9.6)	
Women	1460 (35.3)	13 (0.9)	617 (42.3)	642 (44.0)	188 (12.9)	
Hypertensionª	1584 (55.0)	6 (0.4)	583 (36.8)	756 (47.7)	239 (15.1)	0.12
Diabetes mellitusª	904 (31.4)	5 (0.6)	351 (38.8)	421 (46.6)	127 (14.1)	0.25
Smokingª	114 (4.0)	0 (0.0)	52 (45.6)	49 (43.0)	13 (11.4)	0.16
Chronic kidney diseaseª	140 (4.9)	3 (2.1)	83 (59.3)	46 (32.9)	8 (5.7)	< 0.001
Myocardial infarctionª	54 (1.9)	0 (0.0)	23 (42.6)	26 (48.2)	5 (9.3)	0.57
Previous strokeª	40 (1.4)	0 (0.0)	25 (62.5)	13 (32.5)	2 (5.0)	0.005
Dementiaª	12 (0.4)	0 (0.0)	7 (58.3)	4 (33.3)	1 (8.3)	0.37
Atrial fibrillationª	15 (0.5)	0 (0.0)	5 (33.3)	8 (53.3)	2 (13.3)	1.00
Hospital mortality^§^	747 (18.7)	6 (0.8)	281 (37.6)	342 (45.8)	118 (15.8)	< 0.001
ICU length of stay^¥^	7.4 (10.4)	4.4 (6.0)	6.6 (9.6)	7.7 (10.5)	10.2 (12.8)	< 0.001

For the variables age and ICU length of stay, data are median (IQR).

*Kruskal–Wallis test for continuous and Chi-squared and Fisher’s exact test for categorical variables.

^§^The variable hospital mortality presented 141 missing data.

^¥^The variable ICU length of stay presented 60 missing data.

ªThe variables hypertension, diabetes mellitus, smoking, chronic kidney disease, previous myocardial infarction, previous stroke, dementia, and atrial fibrillation have 1249 missing data.

**Table 2:** Baseline characteristics of study patients aged ≥ 60 years.

Variables	Overall (n = 4053)	BMI categories (kg / m^2^)				p-value*
		< 18.5 (n = 47)	18.5–29.9 (n = 2594)	30.0–39.9 (n = 1264)	≥ 40.0 (n = 148)	
Age (years)	72.0 (13.0)	81.0 (15.0)	73.0 (15.0)	69.0 (11.0)	68.0 (8.5)	< 0.001
**Sex**						< 0.001
Men	2282 (56.3)	23 (1.0)	1567 (68.7)	641 (28.1)	51 (2.2)	
Women	1771 (43.7)	24 (1.4)	1027 (58.0)	623 (35.2)	97 (5.5)	
Hypertensionª	2937 (77.9)	30 (1.0)	1781 (60.6)	995 (33.9)	131 (4.5)	< 0.001
Diabetes mellitusª	1838 (48.7)	17 (0.9)	1093 (59.5)	639 (34.8)	89 (4.8)	< 0.001
Smokingª	192 (5.1)	2 (1.0)	127 (66.2)	57 (29.7)	6 (3.1)	0.80
Chronic kidney diseaseª	408 (10.8)	9 (2.2)	278 (68.1)	110 (27.0)	11 (2.7)	0.01
Myocardial infarctionª	230 (6.1)	4 (1.7)	158 (68.7)	64 (27.8)	4 (1.7)	0.11
Previous strokeª	222 (5.9)	4 (1.8)	177 (79.7)	37 (16.7)	4 (1.8)	< 0.001
Dementiaª	291 (7.7)	17 (5.8)	228 (78.4)	40 (13.8)	6 (2.1)	< 0.001
Atrial fibrillationª	175 (4.7)	3 (1.7)	126 (72.0)	39 (22.3)	7 (4.0)	0.04
Hospital mortality^§^	1738 (43.9)	22 (1.3)	1146 (65.9)	500 (28.8)	70 (4.0)	0.02
ICU length of stay^¥^	8.8 (12.6)	4.7 (10.1)	8.4 (12.2)	9.6 (13.2)	9.1 (12.8)	< 0.001

For the variables age and ICU length of stay, data are median (IQR).

ªThe variables hypertension, diabetes mellitus, smoking, chronic kidney disease, previous myocardial infarction, previous stroke, dementia, and atrial fibrillation have 282 missing data.

*Kruskal–Wallis test for continuous and Chi-squared test for categorical variables.

^§^The variable hospital mortality presented 91 missing data.

^¥^The variable ICU length of stay presented 19 missing data.

For the entire sample (Table 3), stratifying by BMI categories and adjusting for age, sex, and smoking status, those patients with severe obesity showed an increased risk of COVID-19 mortality (HR 1.21; 95% CI 1.03–1.43) compared to those with normal/overweight. No difference was observed for the mild/moderate obesity (HR 0.91; 95% CI 0.83–1.00) and the underweight (HR 1.21; 95% CI 0.80–1.81) categories. In addition, for the survivors in the highest BMI category (≥ 40 kg/m^2^), the length of stay in ICU was 31% higher compared to those in the normal/overweight category (*e*^β^ 1.31; 95% CI 1.17–1.45). An increased length of stay in ICU was also observed for BMI between mild/moderate obesity (*e*^β^ 1.09; 95% CI 1.03–1.16) in comparison to normal/overweight. No difference was detected for the underweight group (*e*^β^ 0.86; 95% CI 0.64–1.15).

**Table 3:** Hazard ratios (HR) for COVID-19 mortality and Length of stay in ICU, according to BMI categories, for the entire sample.

BMI categories	COVID-19 mortality (n = 6474)				Length of stay in ICU (n = 4343)			
	n	HR	95% CI	p-value	n	*e*^β^	95% CI	p-value
Underweight	63	1.21	0.80–1.81	0.37	39	0.86	0.64–1.15	0.30
Normal/overweight	3337	Ref	–	–	2108	Ref	–	–
Mild/moderate obesity	2543	0.91	0.83–1.00	0.06	1830	1.09	1.03–1.16	0.005
Severe obesity	531	1.21	1.03–1.43	0.02	366	1.31	1.17–1.45	< 0.001

Adjusted for age, sex, smoking status.

Table 4 presents the association between BMI categories and in-hospital mortality, according to age groups. For the age group < 60 year, in the unadjusted model (model 1), severe obesity was associated with an increased risk of death (HR 1.27; 95% CI 1.02–1.57) compared to those individuals in the normal/overweight category. After adjusting for the confounders (model 2), the result was similar (HR 1.27; 95% CI 1.01–1.61), and when potential mediator variables were also introduced in the model (model 3), a borderline association was observed (HR 1.25; 95% CI 0.99–1.58). For the underweight category (BMI < 18.5 kg/m^2^), an increased risk for mortality was observed in the crude model (model 1) (HR 2.30; 95% CI 1.03–5.17), and also in model 2, adjusting for confounders (HR 3.74; 95% CI 1.39–10.07), and model 3, after adjusting for confounders and mediator variables (HR 3.71; 95% CI 1.37–9.99). No difference was observed for the mild/moderate obesity category.

**Table 4:** Hazard ratios (HR) for COVID-19 mortality according to age groups and BMI categories.

< 60 years	Model 1 (n = 3989)				Model 2 (n = 2783)				Model 3 (n = 2783)			
	n	HR	95%CI	p-value	n	HR	95%CI	p-value	n	HR	95%CI	p-value
Underweight	24	2.30	1.03–5.17	0.04	18	3.74	1.39–10.07	0.009	18	3.71	1.37–9.99	0.01
Normal/overweight	1731	Ref	–	–	1017	Ref	–	–	1017	Ref	–	–
Mild/moderate obesity	1812	1.12	0.96–1.32	0.15	1353	1.10	0.92–1.32	0.29	1353	1.11	0.92–1.33	0.28
Severe obesity	422	1.27	1.02–1.57	0.03	395	1.27	1.01–1.61	0.04	395	1.25	0.99–1.58	0.06

	Model 1 (n = 3961)				Model 2 (n = 3690)			Model 3 (n = 3690)				
Underweight	47	1.41	0.92–2.15	0.11	45	1.03	0.66–1.61	0.89	45	1.03	0.66–1.60	0.91
Normal/overweight	2532	Ref	–	–	2319	Ref	–	–	2319	Ref	–	–
Mild/moderate obesity	1240	0.82	0.74–0.91	< 0.001	1190	0.87	0.78–0.97	0.02	1190	0.87	0.78–0.97	0.01
Severe obesity	142	1.01	0.79–1.28	0.94	136	1.19	0.93–1.53	0.17	136	1.17	0.91–1.51	0.22

Model 1: unadjusted.

Model 2: adjusted for age, sex, smoking status.

Model 3: adjusted for age, sex, smoking status, hypertension, diabetes.

For those aging ≥ 60 year in the unadjusted analysis (model 1), mild/moderate obesity was associated with a reduced risk of death (HR 0.82; 95% CI 0.74–0.91), compared to the normal/overweight group. After adjusting for the confounders (model 2) and mediation variables (model 3), the reduced mortality risk was still observed (HR 0.87; 95% CI 0.78–0.97, and HR 0.87; 95% CI 0.78–0.97, respectively). No differences were observed for underweight and severe obesity for all three models (Table 4) in comparison to those normal/overweight. Results were similar when considering alternative BMI thresholds among older participants (Table S1 Supplementary Material).

Sensitivity analyses showed similar results when missing data for hypertension, diabetes, and smoking were categorized as cases (model 1), and as non-cases (model 2) (Table S2 Supplementary Material).

Table 5 shows the length of stay in ICU among the survivors, according to age group and BMI categories. For the age group < 60 year, in the unadjusted model, the length of stay in ICU for those patients with severe obesity was 35% higher compared to the normal/overweight category (*e*^β^ 1.35; 95% CI 1.21–1.51). Even after adjustments for confounding (model 2), and potential mediators (model 3), the results were similar (*e*^β^ 1.34; 95% CI 1.19–1.51 and *e*^β^ 1.34; 95% CI 1.19–1.51, respectively). Conversely, for the survivors in the underweight category, the length of stay in ICU was 36% lower compared to the normal/ overweight group, in the unadjusted model (*e*^β^ 0.64; 95% CI 0.42–0.97), and 51% lower after adjusting for confounders (*e*^β^ 0.49; 95% CI 0.31–0.78) and also after adding the mediators in the model (*e*^β^ 0.49; 95% CI 0.31–0.78) (Table 5). For the mild/moderate obesity group, the length of stay in ICU was 7% higher compared to the normal/overweight group (*e*^β^ 1.07; 95% CI 1.00–1.14), although a borderline association was detected. After adjusting for the confounding (model 2) and mediator (model 3) variables, no difference was observed (*e*^β^ 1.05; 95% CI 0.97–1.14 and *e*^β^ 1.06; 95% CI 0.97–1.14, respectively).

**Table 5:** Length of stay in ICU, among the survivors, according to age groups and BMI categories.

< 60 years	Model 1 (n = 3323)				Model 2 (n = 2235)				Model 3 (n = 2235)			
	n	*e*^β^	95% CI	p-value	n	*e*^β^	95% CI	p-value	n	*e*^β^	95% CI	p-value
Underweight	18	0.64	0.42–0.97	0.04	14	0.49	0.31–0.78	0.002	14	0.49	0.31–0.78	0.002
Normal/overweight	1480	Ref	–	–	827	Ref	–	–	827	Ref	–	–
Mild/moderate obesity	1507	1.07	1.00–1.14	0.04	1102	1.05	0.97–1.14	0.20	1102	1.06	0.97–1.14	0.18
Severe obesity	318	1.35	1.21–1.51	< 0.001	292	1.34	1.19–1.51	< 0.001	292	1.34	1.19–1.51	< 0.001

	Model 1 (n = 2296)				Model 2 (n = 2108)				Model 3 (n = 2108)			
Underweight	25	1.13	0.76–1.67	0.55	25	1.23	0.83–1.82	0.31	25	1.23	0.83–1.82	0.31
Normal/overweight	1432	Ref	–	–	1281	Ref	–	–	1281	Ref	–	–
Mild/moderate obesity	761	1.10	1.01–1.20	0.04	728	1.10	1.01–1.21	0.03	728	1.10	1.00–1.21	0.04
Severe obesity	78	1.19	0.95–1.49	0.14	74	1.14	0.90–1.45	0.26	74	1.13	0.89–1.43	0.30

Model 1: unadjusted.

Model 2: adjusted for age, sex, smoking status.

Model 3: adjusted for age, sex, smoking status, hypertension, diabetes.

In the age group ≥ 60 year, the length of stay in ICU for the mild/moderate obesity category was about 10% higher compared to the normal/overweight category, in all three models. However, we did not observe any differences in length of stay in ICU for the survivor’s patients categorized into underweight or severe obesity categories (Table 5).

To investigate the possible influence of the vaccination, a sensitivity analysis excluding those older individuals admitted to ICU between February 01 to May 31, 2021, demonstrated similar results for COVID-19 mortality and length of stay in ICU among the survivors (Tables S3, S4—Supplementary Material).

## Discussion

The present study demonstrated that, in the entire sample, severe obesity (BMI ≥ 40 kg/m^2^) was positively associated with COVID-19 mortality, compared to the normal/overweight category; however, when stratified by age groups, the increased risk of mortality was only observed for the younger category (< 60 years), that also showed an increased risk of death for those in the underweight group. Among the elderly, mild/moderate obesity showed a reduced risk of mortality. For the survivors in the younger group, an increased length of stay in the ICU was observed for those with severe obesity; however, being underweight showed a reduced length of stay in the ICU, when compared to the normal/overweight group. For the survivors in the older group, being mild/moderate obese showed an increased length of stay in the ICU.

Considering that the prevalence of obesity is increasing worldwilde^29^, identifying the groups at higher risk of worse COVID-19 outcomes becomes a priority. In Brazil, according to data from the last National Health Survey, conducted in 2019, the prevalence of obesity is 25.5%, being higher among women (29.5%) when compared to men (21.8%)^30^.

In agreement with our findings, recent systematic reviews^4,5,10,11^ and large cohort studies^31,32^ have demonstrated a positive association between obesity and COVID-19 outcomes. At least five biological mechanisms could explain this association. First, obesity changes the mechanical properties of the lungs and chest wall and can predispose patients to develop respiratory failure in the case of lung infection^33^. Second, obesity can increase the inflammation process which decreases innate and adaptive immunity contributing to worse outcomes in patients with COVID-19^34^. Third, obesity is associated with hypercoagulability, increasing the risk of arterial thrombosis and venous thromboembolism, being one of the most important causes of COVID-19 complication^35^. Fourth, the higher expression of angiotensin-converting enzyme 2 in adipose tissue increases the susceptibility to SARS-CoV-2 infection and the risk of severe disease^36^. Finally, obesity is associated with several comorbidities which are linked with poor COVID-19 outcomes^4,9,37^.

In our study, patients with severe obesity had an increased risk of COVID-19 mortality only for those in the younger group, after adjusting for confounders. Although other studies found an increased mortality risk, a weaker magnitude of the association was also demonstrated for the elderly. In a Spain cohort study, involving approximately 2 million Catalans, the risk of poor COVID-19 outcomes related to increased BMI was higher for those individuals aged ≤ 59 years, compared to the older age group^32^. Another study conducted in the United States also found a similar result, with severe obesity independently associated with COVID-19 mortality (adjusted OR 5.1; 95% CI 2.3–11.1), for the younger population. For the older population, severe obesity was also independently associated with mortality to a lesser extent (adjusted odds ratio 1.6; 95% CI 1.2–2.3)^38^. Finally, a recent Brazilian study^16^, including 313 898 hospitalized patients, observed the mortality risk for obese patients was greater in younger individuals. Gao et al. demonstrated that the mortality risk for each increase in BMI unit decreased progressively, with increasing age becoming non-significant in the 80 years and older age group^15^. Despite few patients, we also observed that being underweight was associated with an increased risk of COVID-19 mortality, among the younger group, as already previously demonstrated^15^.

For the elderly, mild/moderate obesity presented a reduced risk of COVID-19 mortality in our study. Although people with obesity have an increased risk for obesity-related diseases^39^, they present a reduced mortality risk for some respiratory diseases, especially during critical health conditions^20,40^. This situation has been called as “obesity paradox” and might explain how some degree of obesity can provide a protective effect among older patients. According to the paradox hypothesis, a greater fat accumulation provides an additional energy reserve to resist a catabolic environment that usually occurs in ICU^41,42^. Another hypothesis that could explain the obesity paradox is assistance. Older patients with obesity are usually assumed with a poor prognosis, resulting in earlier admission and more aggressive monitoring and management^43^. Finally, elderly patients with obesity are more likely to have hypertension and cardiovascular disease. These comorbidities are commonly treated with angiotensin-converting enzyme inhibitors and angiotensin receptor blockers, decreasing the risk of mortality^44^. Therefore, the “obesity paradox” may also be applied to the COVID-19 scenario.

Consistent findings have already shown an increased risk for ICU admission in obese individuals^5^; however, only a few studies investigated the association between obesity and length of stay in ICU. For this analysis, we included only the survivors, and the results showed that patients with severe obesity in the younger group, and mild/moderate obesity in the older group, had significantly longer ICU length of stay than normal/overweight patients. In the elderly, although the magnitude of the association seems to be greater for the severe obesity patients, it was not statistically significant, probably due to the small sample size in the category. Sjögren and colleagues in Sweden also demonstrated among the survivors that obesity was associated with a doubled risk of ICU length of stay ≥ 14 days, compared to normal BMI group^45^; however, the analysis was not stratified by age groups. This is an important issue because long periods in ICU are related to poor rehabilitation, and increase the risk of opportunistic infection^46^. Also, it demands time from family members, impacts mental health, and promote increased cost for families and the health system^47^. Surprisingly, although patients with underweight showed an increased mortality risk in the younger group, the survivors presented a 51% lower length of stay in ICU, compared to normal/overweight patients.

This study has some limitations. First, body weight and height were self-reported or informed by relatives at the moment of hospital admission. Although this method can lead to measurement error (mainly among older adults due to recall bias), the self-reported method is a valid alternative to determine weight status^48^. Second, some covariates had missing data, which could influence the results; however, we tried to deal with this problem by conducting sensitivity analyses. Third, some variables, identified as a confounder based on the Causal Diagram Theory, may not have been measured in this study and could contribute to residual confounding. Fourth, we do not have access to patients’ clinical conditions, and delays in medical care at the time of admission. Both situations are strongly associated with mortality risk and could influence the results^49^. Fifth, the results of the present study can be generalized only to those individuals who have access to private medical units (most of them with health insurance). However, it could be interpreted as a strength of our work, as most research in Brazil is conducted on public health services. Finally, individuals readmitted to the ICU after hospital discharge were included as new patients; however, according to Todt et al.^50^, the readmission rate is low, and we suppose it did not influence the results.

In summary, our study showed a significant association between severe obesity and an increased risk for COVID-19 mortality and greater length of stay in ICU, in the younger group. Patients with underweight also presented an increased mortality risk; however, the survivors had a reduced length of stay in the ICU. Among the elderly, mild/moderate obesity was associated with reduced COVID-19 mortality risk, corroborating the obesity paradox in this age group, but also showed an increase in length of stay in the ICU. These results could be helpful for public health policy-making, especially in countries affected by a high prevalence of obesity.

## Supplementary Information

Supplementary Tables.

## Data Availability

The dataset used and/or analyzed during the current study is available from the corresponding author on reasonable request. The data collection procedure was performed in accordance with relevant guidelines and regulations.
