# Supplementary material for: Association between obesity and COVID-19 mortality and length of stay in intensive care unit patients in Brazil: a retrospective cohort study
Source: Sci Rep. 2022 Aug 12;12:13737. doi: 10.1038/s41598-022-17197-w (PMC9372981; doi:10.1038/s41598-022-17197-w)
Supplement: Supplementary file 1 — Supplementary Tables. [file 41598_2022_17197_MOESM1_ESM.pdf]

ASSOCIATION BETWEEN OBESITY AND COVID-19 MORTALITY AND LENGTH OF STAY IN INTENSIVE CARE UNIT PATIENTS IN BRAZIL: A RETROSPECTIVE COHORT STUDY

Vitor Barreto Paravidino[1,2*], Tatiana Henriques Leite[1], Mauro Felippe Felix Mediano[3 4], Rosely Sichieri[1], Gulnar Azevedo e Silva[1], Victor Cravo[5], Alex Balduino[6,] Emmanuel Salgueiro[5], Bruno Adler Maccagnan Pinheiro Besen[7], Rodrigo de Carvalho Moreira[6], Carlos Eduardo Brandão[8], Danilo Cosme Klein Gomes[1], Cinthia Almeida Guimarães[1], Pedro Cougo[5]

Table S1. Hazard ratios for COVID-19 mortality and length of stay in ICU for older adults, according to alternative BMI thresholds [1].

**BMI categories**	**COVID-19 mortality (n= 3690)**				**Length of stay in ICU (n= 2108)**			
	n	HR	95%CI	p-value	n	e^{β}	95%CI	p-value
< 22 kg/m^2	257	1.10	0.91-1.35	0.33	147	0.91	0.76-1.09	0.30
22 – 26.9 kg/m^2	1290	ref	---	---	687	ref	---	---
27 - 29.9 kg/m^2	817	0.88	0.77-1.00	0.05	472	1.08	0.96-1.21	0.19
≥ 30 kg/m^2	1326	0.87	0.77-0.97	0.02	802	1.13	1.02-1.25	0.02

adjusted for age, sex, smoking status

Reference

1. Sociedade Española de Nutricion Parenteral y Enteral; SEGG, Sociedad Española de Geriatria y Gerontologia. *Valoración Nutricional Del Anciano*. (1st, ed.).; 2011.

Table S2. Hazard ratios (HR) for COVID-19 mortality, according to age groups and BMI categories, considering the missing data on covariates.

**< 60 y**	**Model 1 (n=3989)**			**Model 2 (n=3989)**		
	HR	95%CI	p-value	HR	95%CI	p-value
underweight	3.00	1.32-6.81	0.009	2.81	1.24-6.37	0.01
normal/overweight	ref	---	---	ref	---	---
mild/moderate obesity	1.13	0.96-1.33	0.14	1.13	0.97-1.33	0.13
severe obesity	1.33	1.07-1.66	0.01	1.28	1.02-1.60	0.03
**≥ 60 y**	**Model 1 (n= 3961)**			**Model 2 (n=3961)**		
	HR	95%CI	p-value	HR	95%CI	p-value
underweight	1.13	0.74-1.72	0.58	1.12	0.73-1.72	0.59
normal/overweight	ref	---	---	ref	---	---
mild/moderate obesity	0.90	0.81-1.00	0.06	0.89	0.80-0.99	0.04
severe obesity	1.19	0.93-1.52	0.17	1.16	0.91-1.49	0.23

Models adjusted for age, sex, smoking status, hypertension, diabetes

Model 1: missing data for hypertension, diabetes, and smoking were categorized as case

Model 2: missing data for hypertension, diabetes, and smoking were categorized as non-case

Table S3. Hazard ratios (HR) for COVID-19 mortality according to age groups and BMI categories, excluding those older individuals admitted to ICU between February 01 to May 31, 2021.

**BMI categories**	**Model 1 (n=2714)**				**Model 2 (n=2533)**				**Model 3 (n=2533)**			
	n	HR	95%CI	p-value	n	HR	95%CI	p-value	n	HR	95%CI	p-value
underweight	38	1.55	1.01-2.40	0.05	36	1.17	0.74-1.84	0.51	36	1.14	0.72-1.81	0.58
normal/overweight	1755	ref	---	---	1614	ref	---	---	1614	ref	---	---
mild/moderate obesity	823	0.81	0.71-0.93	0.002	790	0.89	0.77-1.02	0.09	790	0.89	0.78-1.02	0.10
severe obesity	98	0.86	0.63-1.17	0.34	93	1.05	0.76-1.46	0.77	93	1.04	0.74-1.45	0.83

Model 1: unadjusted

Model 2: adjusted for age, sex, smoking status

Model 3: adjusted for age, sex, smoking status, hypertension, diabetes

Table S4. Length of stay in ICU, among the survivors, according to age groups and BMI categories, excluding those individuals admitted to ICU between February 01 to May 31, 2021.

**BMI categories**	**Model 1 (n=1597)**				**Model 2 (n=1464)**				**Model 3 (n=1464)**			
	n	e^{β}	95%CI	p-value	n	e^{β}	95%CI	p-value	n	e^{β}	95%CI	p-value
underweight	17	1.47	0.90-2.40	0.12	17	1.60	0.98-2.62	0.06	17	1.62	0.99-2.64	0.06
normal/overweight	1009	ref	---	---	904	ref	---	---	904	ref	---	---
mild/moderate obesity	514	1.09	0.98-1.22	0.11	489	1.09	0.97-1.22	0.14	489	1.08	0.96-1.21	0.18
severe obesity	57	1.14	0.87-1.50	0.35	54	1.06	0.80-1.42	0.68	54	1.04	0.78-1.39	0.77

Model 1: unadjusted

Model 2: adjusted for age, sex, smoking status

Model 3: adjusted for age, sex, smoking status, hypertension, diabetes

Table S2. STROBE checklist

		Reporting Item	Page Number
**Title and abstract**			
Title	#1a	Indicate the study's design with a commonly used term in the title or the abstract	1
Abstract	#1b	Provide in the abstract an informative and balanced summary of what was done and what was found	3-4
**Introduction**			
Background / rationale	#2	Explain the scientific background and rationale for the investigation being reported	5-6
Objectives	#3	State specific objectives, including any prespecified hypotheses	6
**Methods**			
Study design	#4	Present key elements of study design early in the paper	6
Setting	#5	Describe the setting, locations, and relevant dates, including periods of recruitment, exposure, follow-up, and data collection	6-7
Eligibility criteria	#6a	Give the eligibility criteria, and the sources and methods of selection of participants. Describe methods of follow-up.	7
Eligibility criteria	#6b	For matched studies, give matching criteria and number of exposed and unexposed	N/A
Variables	#7	Clearly define all outcomes, exposures, predictors, potential confounders, and effect modifiers. Give diagnostic criteria, if applicable	7-8
Data sources / measurement	#8	For each variable of interest give sources of data and details of methods of assessment (measurement). Describe comparability of assessment methods if there is more than one group. Give information separately for exposed and unexposed groups if applicable.	7-8
Bias	#9	Describe any efforts to address potential sources of bias	9
Study size	#10	Explain how the study size was arrived at	7

Quantitative variables	#11	Explain how quantitative variables were handled in the analyses. If applicable, describe which groupings were chosen, and why	8
Statistical methods	#12a	Describe all statistical methods, including those used to control for confounding	8-9
Statistical methods	#12b	Describe any methods used to examine subgroups and interactions	8-9
Statistical methods	#12c	Explain how missing data were addressed	8-9
Statistical methods	#12d	If applicable, explain how loss to follow-up was addressed	N/A
Statistical methods	#12e	Describe any sensitivity analyses	9
**Results**			
Participants	#13a	Report numbers of individuals at each stage of study—eg numbers potentially eligible, examined for eligibility, confirmed eligible, included in the study, completing follow-up, and analysed. Give information separately for for exposed and unexposed groups if applicable.	9-10
Participants	#13b	Give reasons for non-participation at each stage	9-10
Participants	#13c	Consider use of a flow diagram	10
Descriptive data	#14a	Give characteristics of study participants (eg demographic, clinical, social) and information on exposures and potential confounders. Give information separately for exposed and unexposed groups if applicable.	Table 1/2
Descriptive data	#14b	Indicate number of participants with missing data for each variable of interest	Table 1/2 (legend)
Descriptive data	#14c	Summarise follow-up time (eg, average and total amount)	Table 1/2
Outcome data	#15	Report numbers of outcome events or summary measures over time. Give information separately for exposed and unexposed groups if applicable.	Table 1/2
Main results	#16a	Give unadjusted estimates and, if applicable, confounder-adjusted estimates and their precision (eg, 95% confidence interval). Make clear which confounders were adjusted for and why they were included	Table 3/4/5
Main results	#16b	Report category boundaries when continuous variables were categorized	Table 3/4/5

Main results	#16c	If relevant, consider translating estimates of relative risk into absolute risk for a meaningful time period	N/A
Other analyses	#17	Report other analyses done—eg analyses of subgroups and interactions, and sensitivity analyses	Table S1/2/3/4 suppl
**Discussion**			
Key results	#18	Summarise key results with reference to study objectives	18
Limitations	#19	Discuss limitations of the study, taking into account sources of potential bias or imprecision. Discuss both direction and magnitude of any potential bias.	20-21
Interpretation	#20	Give a cautious overall interpretation considering objectives, limitations, multiplicity of analyses, results from similar studies, and other relevant evidence.	20-21
Generalisability	#21	Discuss the generalisability (external validity) of the study results	21
**Other Information**			
Funding	#22	Give the source of funding and the role of the funders for the present study and, if applicable, for the original study on which the present article is based	21

Reference:

von Elm E, Altman DG, Egger M, Pocock SJ, Gotzsche PC, Vandenbroucke JP. The Strengthening the Reporting of Observational Studies in Epidemiology (STROBE) Statement: guidelines for reporting observational studies
